# The effects of gratitude interventions: a systematic review and meta-analysis

**DOI:** 10.31744/einstein_journal/2023RW0371

**Published:** 2023-07-31

**Authors:** Geyze Diniz, Ligia Korkes, Luca Schiliró Tristão, Rosangela Pelegrini, Patrícia Lacerda Bellodi, Wanderley Marques Bernardo

**Affiliations:** 1 Plenae São Paulo SP Brazil Plenae, São Paulo, SP, Brazil.; 2 Faculdade de Ciências Médicas de Santos Santos SP Brazil Faculdade de Ciências Médicas de Santos, Santos, SP, Brazil.; 3 São Paulo SP Brazil Psychologist, São Paulo, SP, Brazil.; 4 Faculdade de Medicina Universidade de São Paulo São Paulo SP Brazil Faculdade de Medicina, Universidade de São Paulo, São Paulo, SP, Brazil.

**Keywords:** Gratitude, Depression, Anxiety, Mental health, Emotions, Health facilities, Delivery of health care, Personnel satisfaction, Narration

## Abstract

**Introduction:**

Gratitude has several implications. Over time, a logical relationship has been established between gratitude and well-being. In addition, researchers aimed to establish associations between gratitude and other factors of positive feelings using scientific methods. We conducted a systematic review and meta-analysis of interventions to develop gratitude and its benefits to human beings.

**Objective:**

This study aimed to evaluate and quantify the available scientific evidence on interventions to acquire knowledge on gratitude as a quantifiable causal factor of benefit to human beings.

**Methods:**

A systematic literature search was conducted to identify studies that investigated the effects of gratitude interventions. MEDLINE, Embase, and Central Cochrane databases were searched in addition to gray (Google Scholar) and manual search. Two authors independently evaluated the titles and abstracts, and selected the studies that met the inclusion criteria. The searches were conducted between January and July 2022.

**Results:**

Sixty-four randomized clinical trials were included. The meta-analysis demonstrated that patients who underwent gratitude interventions experienced greater feelings of gratitude, better mental health, and fewer symptoms of anxiety and depression. Moreover, they experienced other benefits such as a more positive mood and emotions.

**Conclusion:**

The results demonstrate that acts of gratitude can be used as a therapeutic complement for treating anxiety and depression and can increase positive feelings and emotions in the general population. Prospero database registration: (www.crd.york.ac.uk/prospero) under the number CRD42021250799.

## INTRODUCTION

Gratitude is difficult to define. It has been conceptualized as an emotion, attitude, moral virtue, habit, personality trait, and coping response.^(1)^ It appears to be related to personality traits as well as subjective and moral well-being.

Gratitude is a light expression not necessarily conditioned to good times, making it possible to maintain the feeling and feel good, even during negative experiences or most difficult moments.

In its definition, elements such as grace, presence, love, health, food, nature, beauty, and life have been recognized, which can be reflected in a state of fulfillment while enjoying and valuing the trajectory more than the result itself.

Logical relationship has been established between gratitude and well-being, with the idea that gratitude fosters positive feelings that contribute to a general sense of well-being. In addition, researchers have sought to establish other associations with factors of positive feelings using scientific methods.

Methods for measuring the level of gratitude, for diagnosis and prognostic impact, have been developed and validated through questionnaires administered in different ethnicities, languages, or countries, such as the Gratitude Questionnaire-Six-Item Form (GQ-6) of McCullough et al.^(2)^ and the Gratitude Resentment and Appreciation Test (GRAT).^(3)^ GQ-6 was correlated positively with optimism, life satisfaction, hope, spirituality/religiosity, forgiveness, empathy, and prosocial behavior, and negatively with depression, anxiety, materialism, and envy.^(4)^

Interventions to stimulate, develop, and acknowledge feelings of gratitude have been tested by evaluating their impact on beneficial outcomes through randomized clinical trials. These outcomes include those related to quality of life, well-being, health, aging, positive and/or negative feelings, and social behavior.

## OBJECTIVE

To evaluate and quantify the scientific evidence on gratitude, we conducted a systematic review and meta-analysis of interventional studies on development of gratitude and its benefits to human beings.

## METHODS

This systematic review followed the Preferred Reporting Items for Systematic Reviews and Meta-Analyses (PRISMA) guidelines^(5)^ and the details of the protocol are registered in the International Prospective Register of Systematic Reviews (PROSPERO).^(6)^

We searched the MEDLINE, Embase, and Central Cochrane databases. In addition, gray (Google Scholar) and manual searches were conducted. The terms (“Grateful” OR “Gratitude” OR “Gratefulness”) were searched in the titles, abstracts, and keywords. The searches were conducted between January and July 2022.

The eligibility criteria for the studies were as follows: (I) children, teenagers, adults, or older adults; (II) interventions to acquire the concept or practice of gratitude; (III) measures of association, correlation, or effect related to beneficial or harmful outcomes; (IV) randomized clinical trials; (V) no period restriction; (VI) languages: English, Spanish, Portuguese, or Italian; and (VII) full-text or abstract with relevant data is available.

Two authors independently evaluated the titles and abstracts of the studies identified in the search, and those meeting the inclusion criteria were selected for review. In cases of disagreement, a third author was consulted for resolving the issue.

The following data were extracted from the selected studies: name, year of publication, population, description of the intervention or exposure, measurement method or definition of the presence or absence of gratitude, outcome of benefit or harm, and length of follow-up.

The outcomes analyzed were directly related to the focus of the selected evidence and varied between gratitude development, life satisfaction, mental health, anxiety and depression symptoms, sleep-related outcomes, positive and negative affect, positive feelings, emotions, and attitudes, and negative feelings, emotions, and attitudes.

Mean score, standard deviation, and mean difference were used to express the outcomes. For categorical variables, the measures were absolute score, percentages, risk differences, and the score needed for positive or negative outcome. The level of significance was 95%. The measures to assess gratitude were related to the scores used.

The risk of bias was assessed using the Risk of Bias 2 (RoB 2)^(7)^ tool for interventional studies and was classified as low, high, or very high.

For the meta-analysis, Review Manager (RevMan) Version 5.4^(8)^ was used. Comparisons were presented in terms of mean difference (MD) and 95% confidence interval (CI). The inconsistency in the effects of the interventions was assessed using I^2^. The random effects and fixed effects models were used if I^2^ >50% and I^2^ ≤50%, respectively. We used a funnel plot for asymmetry to assess the possible publication bias.

The certainty of evidence was assessed using the GRADE Pro Guideline Development Tool^(9)^ and was classified as high, moderate, low, or very low (Appendix A).

## RESULTS

### Study selection

**Figure 1:**
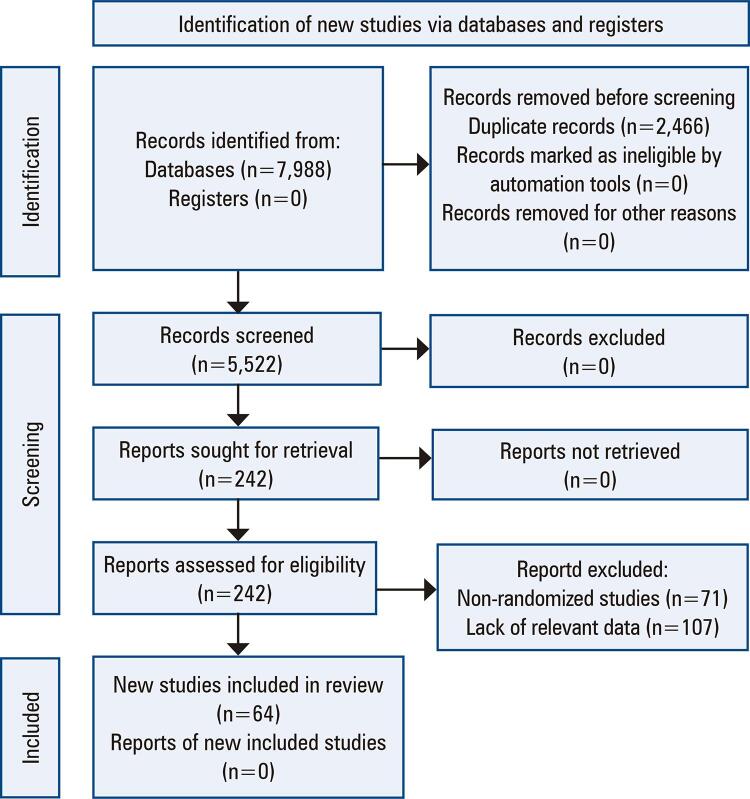
PRISMA flow diagram

A total of 5,522 articles were retrieved after the removal of duplicates. Of these, 1,365 titles and abstracts were selected and evaluated for eligibility, of which 242 were selected for full-text evaluation. Finally, 64 articles^(1,10-72)^met the eligibility criteria (Appendix B) and were included in the systematic review and 33 in the meta-analysis (Figure 1).

### Overview

The selected studies (Appendix B) were published between 2003 and 2021, had sample sizes from 23 to 1,337, and included children with depression, teenagers, adults, and older adults as participants. The gratitude interventions used in the studies varied between gratitude diaries, conversation programs, training, and visits, expression of gratitude to others (verbally or in writing), publishing pictures with captions of gratitude, and thinking of things that makes one feel grateful. The main interventions for the control groups in the selected articles were writing food or normal diaries, staying on a waiting list, thinking about or performing daily activities, not performing any activities, and completing questionnaires. Most studies were classified as having a high risk of bias (Appendix C).

### Meta-analysis

#### Gratitude

Three different scores indicated greater gratitude in the groups that underwent gratitude interventions.

**Figure 2:**
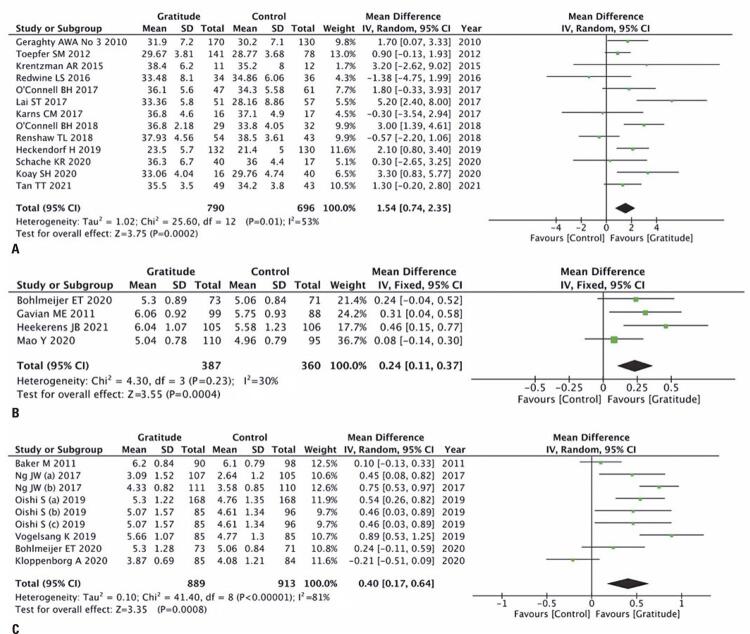
Gratitude forest plot. 2A) Total GQ-6; 2B) Mean GQ-6; 2C) Numerical gratitude scale ranging from 1 to 7

SD: standard deviation; IV: inverse variance; 95%CI: 95% confidence interval.

Thirteen articles involving 1,486 patients applied the GQ-6, and the meta-analysis showed that the score was 3.67% higher in participants who underwent gratitude intervention (MD= 1.54; 95%CI= 0.74, 2.35; p=0.0002; I^2^=53%; random effects; certainty: very low) (Figure 2A). Four other articles reported the mean GQ-6 score, which was also superior in the gratitude group, with a 3.42% benefit (MD= 0.24; 95%IC= 0.11, 0.37; p=0.004; I^2^=30%; fixed effect; certainty: low) (Figure 2B).

In addition to the GQ-6, the meta-analysis of nine articles (n=1,802) that applied a numerical gratitude scale ranging from 1 to 7 showed a 5.7% higher score for the group that underwent gratitude interventions (MD= 0.40; 95%CI= 0.17, 0.64; p=0.0008; I^2^=81%, random effects; certainty: very low) (Figure 2C).

## Satisfaction with life

**Figure 3:**
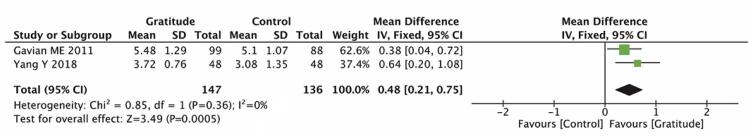
Satisfaction With Life Scale forest plot

SD: standard deviation; IV: inverse variance; 95%CI: 95% confidence interval.

Two studies (n=283) reported mean Satisfaction With Life Scale (SWLS) scores.^(73)^ The analysis showed that there was greater satisfaction in patients who underwent gratitude interventions, with a 6.86% higher score (MD= 0.48; 95%CI= 0.21, 0.75; p=0.005; I^2^=0%, fixed effect; certainty: low) (Figure 3).

## Mental health

**Figure 4:**
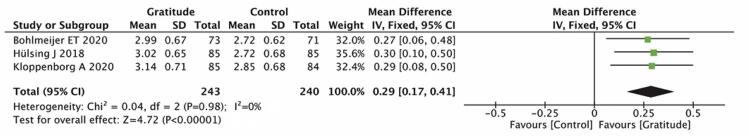
Mental Health Continuum - Short Form forest plot

SD: standard deviation; IV: inverse variance; 95%CI: 95% confidence interval.

Mental health was assessed in three articles (n=483) using the Mental Health Continuum-Short Form (MHC-SF).^(74)^ The results showed that the average score was 5.8% higher in patients who underwent gratitude interventions (MD= 0.29; 95%CI= 0.17, 0.41; p<0.00001; I^2^=0%, fixed effect; certainty: low) (Figure 4).

## Anxiety

**Figure 5:**
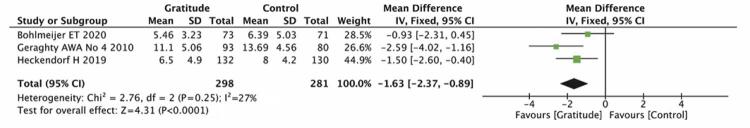
Generalized Anxiety Disorder score forest plot

SD: standard deviation; IV: inverse variance; 95%CI: 95% confidence interval.

The analysis of 579 patients in three articles showed that gratitude interventions led to fewer anxiety symptoms, with a 7.76% lower Generalized Anxiety Disorder (GAD-7)^(75)^ score than that of the control group (MD= -1.63; 95%CI= -2.37, -0.89; p<0.0001; I^2^=27%, fixed effect; certainty: low) (Figure 5).

## Depression

**Figure 6:**
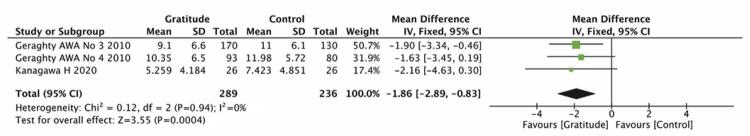
Patient Health Questionnaire-9 forest plot

SD: standard deviation; IV: inverse variance; 95%CI: 95% confidence interval.

Depression symptoms (n=525) were assessed in three articles using the Patient Health Questionnaire-9 (PHQ-9).^(76)^ Analysis of the results showed that patients who underwent gratitude interventions had fewer symptoms of depression with 6.89% lower score than that of the control group (MD= -1.86; 95%CI= -2.89, -0.83; p<0.0004; I^2^=0%; fixed effect; certainty: low) (Figure 6).

## Qualitative analysis

The qualitative outcomes of the selected articles demonstrated several benefits for the participants who underwent gratitude interventions.

Ducasse et al.^(19)^ showed that the intervention group was more optimistic (p=0.01). Oishi et al.^(49)^ (students with an average age of 20 years and 18 years: p=0.03 and p=0.05, respectively) and DeSteno et al.^(18)^ (p<0.00001) showed that participants in the gratitude groups had greater appreciation. DeSteno et al.^(18)^ found more positive emotions in the gratitude group (p<0.0001). Ng et al.^(46)^ demonstrated greater positive mood in patients who underwent gratitude interventions. Finally, Grant et al.^(23)^reported that participants in the gratitude group exhibited more prosocial behaviors.

Heckendorf et al.^(25)^ applied the Penn State Worry Questionnaire,^(77)^ which assesses worry of participants, and demonstrated that those who underwent gratitude interventions had a lower score (p=0.009). Ducasse et al.^(19)^ assessed psychological pain and reported lower scores in patients in the intervention group (p=0.05).

In the happiness outcome, two scores (Subjective Happiness Scale^(78)^ and Authentic Happiness Inventory^(79)^) were not significantly different, while two numerical scales (1–5 and 1–7) showed benefits for the gratitude groups. When analyzing sleep-related outcomes, only the Insomnia Severity Index^(80)^ showed a significant difference in favor of the gratitude group, whereas analysis of sleep quality and the Pittsburgh Sleep Quality Index^(81)^ showed no difference. Moreover, there was no significant difference in any positive and negative affect scores (Implicit Positive and Negative Affect Test,^(82)^ Scale of Positive and Negative Experience,^(73)^ Positive Affect Negative Affect Scale,^(83)^ and a numerical scale of 1–5), except in the Affect Balance Scale^(84)^ reported by Yang et al.^(72)^ that showed higher positive affect (p<0.0001) and lower negative affect (p=0.02) in the gratitude group.

## DISCUSSION

This systematic review and meta-analysis demonstrated that participants who underwent gratitude interventions had greater feelings of gratitude (up to 4% higher scores), greater satisfaction with life (6.86% higher), better mental health (5.8% higher), and fewer symptoms of anxiety and depression (7.76% and 6.89% lower scores, respectively). Moreover, they had other benefits such as more positive moods and emotions, greater appreciation and optimism, more prosocial behavior, less worry, and less psychological pain.

### The virtue of being grateful

Although the science of psychology began to pay attention to gratitude in terms of clinical research only approximately 20 years ago, gratitude is inborn to humans and is a source of emotional balance and well-being and impacts interpersonal relationships.

The exercise of gratitude is considered a strong therapeutic tool for positive psychology in obtaining responses that combat disorders and other issues related to depression and anxiety. In addition to being a psychotherapeutic instrument, gratitude is considered essential for forming the personality and character of an individual.

When expressing gratitude, people avoid pessimism, unhappiness, complaints of malaise and pain, toxic emotions such as anger, hurt, and fear, feelings of loneliness, isolation, and lack of engagement. A grateful individual focuses on positive practices of solidarity and attention to others and gains a sense of well-being in return.

However, being grateful, that is, expressing gratitude, is difficult for many people. They do not understand the importance of developing a thankful spirit. Psychotherapy and interventions of sensibilization and emotional education can assist these people in understanding the importance of “being grateful” and exercising this virtue.

As this occurs, positive changes in emotional health are experienced by the individual and perceived by others.

### Gratitude: anxiety and depression

A relevant finding of this review was the improvement in anxiety and depression symptoms in patients who underwent gratitude interventions. Although it had a small effect compared to other therapies, such as medications, stimulating gratitude can complement other therapies in these patients.

### Applicability

Gratitude intervention is accessible and easy to implement. Several applications offer functionalities for users to describe what makes them feel grateful, working as a diary or an intervention, as seen in the included articles. Stimulating the expression of gratitude toward another person verbally or in writing is another easily implemented practice. Additionally, as demonstrated by Koay et al.^(38)^ posting pictures on social media with captions of gratitude could also be a way to express gratitude. Finally, encouraging the simple act of thinking about gratitude benefits people. This application can help in interventions not only for patients but also for the general population.

The increase in positive feelings can reverberate throughout a complex chain of neurotransmitters, capable of not only perpetuating a sense of well-being but also acting chemically in this regard.

This study identified the relationship between gratitude and reduction of anxiety and depression, which are relevant everyday emotional comorbidities that affect individuals’ quality of life. Psychiatric illnesses tend to be chronic, require intensive treatment, and have other organic consequences. If practicing gratitude-a simple act that can be performed throughout the day at no cost-can minimize psychiatric illnesses, its implementation should be a priority. Quality of life is the macro subject of our concerns, and gratitude is the feeling that can favor living fully by increasing satisfaction with life, mental health, and obtaining positive feelings.

### Participants

The selected articles included a wide range of participants such as patients with neuromuscular diseases, prisoners, children, adolescents, adults, and doctors. We consider this heterogeneity of the participants as a strong point of the review, as it shows the positive impacts of developing gratitude throughout life and in different contexts. For example, the emotional state and context of someone with a serious illness and those of college students differ significantly. Nevertheless, our results showed a clear benefit to the groups that underwent gratitude interventions, proving that gratitude can benefit people in different contexts, cultures, ages, professions, and health statuses.

### Limitations

Although these results are relevant, they must be analyzed cautiously. The high heterogeneity between the methodologies of the studies may have affected the results and the certainty of the evidence. There was great diversity among gratitude interventions. The lack of blinding, high loss of follow-up of the participants, and the analysis by protocol instead of intention-to-treat in some studies led to all outcomes being classified as having a high risk of bias and impacted the certainty of evidence.

### Strengths

This systematic review adds unprecedented information to the literature. Despite the existence of several scores for quantifying subjective outcomes such as those related to emotions and feelings, statistical expression of the effect size and precision of these outcomes are still not routine, particularly in the form of meta-analyses. Quantifying outcomes and analyzing the certainty of evidence with proper tools are paramount for generating evidence. These findings highlight the importance of this study. This is the first systematic review and meta-analysis to compare the outcomes of gratitude interventions exclusively with control groups. This eliminated possible confounding factors, as opposed to the articles that included comparison groups that underwent hassle or happiness interventions. In addition, the principles of systematic reviews and meta-analyses aim to increase the power of conclusions by synthesizing results of several studies on the same outcome and increasing the ability (by increasing the number of samples analyzed) to identify effects, even if there is little benefit to individuals. Furthermore, by identifying the important outcomes measured by various studies, we were able to highlight not only the ways to measure them but also their specific clinical importance. Moreover, most of the articles in this review were retrieved from gray literature, usually not covered by other reviews, including some that were never included in any review. Finally, there was great diversity in participant characteristics, as articles included teenagers, physicians, college students, prisoners, older adults, patients with neuromuscular diseases, and children with depression (Appendix B). Including various types of populations in the analysis can be considered another advantage of systematic reviews and meta-analyses.

### Future studies

Future studies must use an appropriate methodology to correct the biases that we found to have a greater level of certainty in evidence. It is necessary to have homogeneous methodologies with comparable interventions, blinding of evaluators and patients, and longer follow-ups. In addition, the scores and questionnaires used must be standardized. A major difficulty in this review was the great variability in the scores used in the studies. If future studies use a uniform methodology, the results will be more accurate and reliable. In terms of quality of life, it is necessary to remember the importance of balancing other components such as the body, mind, spirituality, relationships, purpose, and context, which will certainly be included in an ongoing research project through scientific models and systematic reviews.

## CONCLUSION

This meta-analysis revealed that developing feelings and performing acts of gratitude are related to a greater sense of gratitude and satisfaction with life, better mental health, and fewer symptoms of anxiety and depression. Furthermore, qualitative analysis demonstrated other benefits such as more positive emotions and moods, greater appreciation and optimism, more prosocial behavior, less worry, and less psychological pain. The results demonstrate that developing feelings and performing acts of gratitude can be used as a therapeutic complement in treating anxiety and depression, and can increase positive feelings and emotions in the general population.
