# Supplementary material for: The effects of gratitude interventions: a systematic review and meta-analysis
Source: Einstein (Sao Paulo). 2023 Jul 31;21:eRW0371. doi: 10.31744/einstein_journal/2023RW0371 (PMC10393216; doi:10.31744/einstein_journal/2023RW0371)
Supplement: Supplementary file 1 [file 2317-6385-eins-21-eRW0371-suppl01.pdf]

Appendix A. GRADEPro certainty of evidence table

Certainty assessment							Number of patients		Effect		Certainty
Number of studies	Study design	Risk of bias	Inconsistency	Indirectness	Imprecision	Other considerations	Gratitude	Control	Relative (95%CI)	Absolute (95%CI)	
GQ-6 total score											
13	Randomized trials	Very serious[a]	Serious[b]	Not serious	Not serious	None	790	696	-	MD 1.54 higher (0.74 higher to 2.35 higher)	⨁◯◯◯ Very low
GQ-6 mean score											
4	Randomized trials	Very serious[a]	Not serious	Not serious	Not serious	None	387	360	-	MD 0.24 higher (0.11 higher to 0.37 higher)	⨁⨁◯◯ Low
Gratitude (numerical scale 1–7)											
9	Randomized trials	Very serious[a]	Very serious[c]	Not serious	Not serious	None	889	913	-	MD 0.4 higher (0.17 higher to 0.64 higher)	⨁◯◯◯ Very low
SWLS mean score											
2	Randomized trials	Very serious[a]	Not serious	Not serious	Not serious	None	147	136	-	MD 0.48 higher (0.21 higher to 0.75 higher)	⨁⨁◯◯ Low
GAD-7											
3	Randomized trials	Very serious[a]	Not serious	Not serious	Not serious	None	298	281	-	MD 1.63 lower (2.37 lower to 0.89 lower)	⨁⨁◯◯ Low
MHC-SF											
3	Randomized trials	Very serious[a]	Not serious	Not serious	Not serious	None	243	240	-	MD 0.29 higher (0.17 higher to 0.41 higher)	⨁⨁◯◯ Low
PHQ-9											
3	Randomized trials	Very serious[a]	Not serious	Not serious	not serious	none	289	236	-	MD 1.86 lower (2.89 lower to 0.83 lower)	⨁⨁◯◯ Low

95%CI: 95% confidence interval; MD: mean difference; PHQ-9: Patient Health Questionnaire; MHC-SF: Mental Health Continuum-Short Form; GAD-7: Generalized Anxiety Disorder; SWLS: Satisfaction With Life Scale; GQ-6: Gratitude Questionnaire-Six-Item Form.
Explanations:
a. Studies did not blind participants or evaluators; losses >20%; protocol analysis.
b. I^2 between 50-75%.
c. I^2 >75%.

Appendix B. Study characteristics

Author	Population	Type of intervention	Intervention	Comparison	Outcomes	Follow-up
Emmons et al.[1]	Students (n=196)	Isolated	Gratitude intervention (n=65)	Hassles (n=64) and control (n=67)	Well-being; gratitude; physical symptoms; gratitude	9 weeks
	Students (n=157)	Isolated	Gratitude intervention (n=52)	Hassles (N=49) and control (n=56)	Health and prosocial behavior	2 weeks
	Patients with neuromuscular diseases (n=65)	Isolated	Gratitude intervention (n=33)	Control (n=32)	Affect; well-being; health behavior; daily activities	3 weeks
Antoine et al.[10]	Couples (n=76)	Combined	Positive psychology intervention (n=40)	Control (n=36)	Satisfaction (SWLS)	1 month
Baker et al.[11]	Workers (n=188)	Isolated	Gratitude intervention (n=90)	Control (n=98)	Gratitude (numeric scale); affect (PANAS); well-being (numeric scale); satisfaction; commitment	5 weeks
Bartlett et al.[12]	Adults with an average age of 73 years (n=42)	Isolated	Daily gratitude exercise (n=23)	Only surveys completions (n=19)	Gratitude (GQ-6); solitude (PANAS); subjective well-being; subjective health; symptoms; daily activities; general positivity	3 weeks
Bohlmeijer et al.[13]	People with moderate / low well-being and moderate depression and anxiety (n=217)	Isolated	Gratitude exercises (n=73)	Self-love act (n=73) Waiting list (n=71)	Well-being (MHC-SF); depression (CES-D); anxiety (GAD-7); gratitude (GQ-6, numeric scale)	6 months
Cheng et al.[14]	Health professionals (n=102)	Isolated	Gratitude diary (n=34)	Hassle diary (n=34) or no treatment (n=34)	Depression (CES-D); stress (PSS)	3 months
Cunha et al.[15]	Adults with an average age of 32 years (n=1,337)	Isolated	Gratitude stimulation (n=446)	Hassles (n=444) and neutral (n=447)	Affect (PANAS); depression (CES-D); happiness (SHS); satisfaction (SWLS)	4 weeks
Datu et al.[16]	Students (n=86)	Isolated	List 5 things you are grateful (n=43)	Weekly diary (n=43)	Positive and negative emotions; life satisfaction	3 weeks
Dennis et al.[17]	Participants during lockdown (n=216)	Isolated	Gratitude intervention (n=67)	Best possible self (n=73) Nostalgia (n=63) Control (n=58)	Affect (PANAS)	No follow-up
DeSteno et al.[18]	Adults with an average age of 19 years (n=75)	Isolated	Gratitude stimulation (n=25)	Induction of happiness (n=25) and neutral (n=25)	Gratitude and happiness	No follow-up
Ducasse et al.[19]	Patients hospitalized for suicidal attempt or ideation (n=201)	Isolated	Gratitude writing (n=101)	Food diary (n=100)	Psychological pain (numeric scale); severity and intensity of suicidal ideation (C-SSRS; SSI; numeric scale); hope and optimism; depression and anxiety (BDI)	1 weeks
Froh et al.[20]	Students (n=89)	Isolated	Gratitude intervention (n=44)	Control (n=45)	Gratitude (GAC); affect (PANAS)	2 months
Gavian et al.[21]	Students (n=271)	Isolated	Gratitude intervention (n=99)	Relaxation intervention (n=84) and control (n=88)	Gratitude (GQ-6); relaxation (SRSI); affect (PANAS); satisfaction (SWLS); stress (PCOSES-8, DSI); serenity (SS); depression (DASS-21); physical symptoms (CHIPS); events gravity (SES)	1 month
Geraghty et al.[22]	Participants (n=447)	Isolated	Gratitude diary (n=170)	Automatic thinking (n=147) Waiting list (n=130)	Loss of adhesion; depression (PHQ-9); automatic thinking (ATQ); satisfaction (SWLS); affect (PANAS); health control (MHLC); expectation	2 weeks
	Participants (n=247)	Isolated	Gratitude diary (n=93)	Worry (n=74) Waiting list (n=80)	Loss of adhesion; worry (PSWQ-PW); anxiety (GAD-7); depression (PHQ-9); hope (AHS); optimism (LOT -R); expectation; auto-control	2 weeks

continue...

...Continuation

Appendix B. Study characteristics

Author	Population	Type of intervention	Intervention	Comparison	Outcomes	Follow-up
Grant et al.[23]	Students (n=69)	Isolated	Gratitude intervention (n=35)	Control (n=34)	Prosocial behavior, self-efficacy, social function, affect, gratitude	No follow-up
	Students (n=57)	Isolated	Gratitude intervention (n=29)	Control (n=28)		
	Fundraisers (n=41)	Isolated	Gratitude intervention (n=20)	Control (n=21)	Prosocial behavior, self-efficacy, social function	
	Students (n=79)	Isolated	Gratitude intervention (n=40)	Control (n=39)	Prosocial behavior, self-efficacy, social function, gratitude	
Gulliford et al.[24]	Participants (n=52)	Isolated	Gratitude intervention (n=25)	Pride (n= 25)	Gratitude (GRAT-S, MCGM)	No follow-up
Heckendorf et al.[25]	Adults with an average age of 42 years (n=262)	Isolated	Gratitude training based on smartphone usage and online (n=132)	Waiting list (n=130)	Negative thoughts (PTQ); depression (CES-D); anxiety (GAD-7); resilience (CD-RISC); insomnia (ISI); worry (PSWQ); social support (BSSS); gratitude (GQ-6); optimism (LOT-R); satisfaction (CSQ-I)	6 weeks; 3 and 6 months
Heekerens et al.[26]	Adults with an average age of 43 years (n=211)	Isolated	Letter of interpersonal gratitude (n=105)	Weekly diary (n=106)	Gratitude (GQ-6)	No follow-up
Heintzelman et al.[27]	Adults with an average age of 49 years (n=55)	Combined	Face-to-face ENHANCE (central self, experiential self, social self, reflection, person-activity adequacy and development of habits) (n=27)	Waiting list (n=28)	Subjective well-being (SWB); life satisfaction (SWLS and scales); life events (PNMT); psychological health (depression and stress)	3 months
	Adults with an average age of 43 years (n=100)	Combined	Online ENHANCE (n=50)	Waiting list (n=50)		
Hirshberg et al.[28]	Students (n=162)	Isolated	Gratitude stimulation (n=41)	Breathing exercise (n=34); stimulating kindness and love (n=42); no intervention (n=39)	Anxiety (ASI); gratitude (GQ-6); affect (IPANAT, PANAS); stress	No follow-up
Ho et al.[29]	Clusters (n=1,261)	Combined	Positive psychology intervention: joy, gratitude, flow, taste and listen (n=828)	Control (n=433)	Happiness (SHS), quality of life (SF-12v2)	12 weeks
		Combined			Family communication (FCS); health, happiness and family harmony	
Hülsing et al.[30]	Participants (n=170)	Isolated	Gratitude intervention (n=85)	Waiting list (n=85)	Gratitude (GRAT-S); well-being (MHC-SF)	12 weeks
Hussong et al.[31]	Parents with children between 6 and 9 years (n=104)	Isolated	Gratitude conversation program (n=53)	Waiting list (n=51)	Clarity and satisfaction; behavioral intentions; parental trust; knowledge consumption; socialization behaviors; children's gratitude; father-son gratitude and missed opportunities.	1 month
Jackowska et al.[32]	Women with sleep and emotional disorders (n=119)	Isolated	Gratitude writing (n=40)	Reporting of daily events (n=41) and no treatment (n=38)	Well-being (SWB; PESS; HADS; FS; RLOT); sleep (PSQI; quality)	3 weeks
Kanagawa et al.[33]	College students (n=79)	Isolated	Letter of interpersonal gratitude (n=26)	Setting self-help goals and planning intervention (n=26) Control (n=26)	Depression (PHQ-9)	4 weeks
Karns et al.[34]	Students (n=33)	Isolated	Gratitude intervention (n=16)	Control (n=17)	Gratitude (GQ-6); altruism (principles of care)	3 weeks
Ki et al.[35]	Health professionals (n=180)	Isolated	Gratitude intervention (n=90)	Hassle (n=90)	Stress (PSS); gratitude (GQ-6); affect (CAS); satisfaction (SWLS); depression (CES-D 10);	1 week
Kini et al.[36]	Psychotherapy patients (n=43)	Isolated	Gratitude diary (n=22)	Psychotherapy (n=21)	Gratitude (GQ-6; GAC3; BHM-20)	12 weeks
Kloppenborg et al.[37]	Participants (n=169)	Isolated	Gratitude intervention (n=85)	Waiting list (n=84)	Well-being (MHC-SF); gratitude (numeric scale)	6 weeks
Koay et al.[38]	College students (n=33)	Isolated	Post pictures with gratitude captions (n=16)	Control (n=17)	Gratitude (GQ-6); stress (PSS); satisfaction (SWLS)	1 week

continue...

...Continuation

Appendix B. Study characteristics

Author	Population	Type of intervention	Intervention	Comparison	Outcomes	Follow-up
Kobayashi et al.[39]	College students (n=66)	Isolated	Think 3 things that make feel grateful to parents (n=22) Express gratitude to parents (n=22)	Control (n=22)	Happiness (SHS); satisfaction (SWLS)	1 week
Krentzman et al.[40]	Adults with alcohol abuse (n=23)	Isolated	Gratitude intervention (n=11)	Control (n=12)	Feasibility and acceptance; affect (PANAS); gratitude (GQ-6); cognition	8 and 14 weeks
Kwok et al.[41]	Children with depression (n=68)	Combined	Sessions of gratitude and hope (n=34)	Control (n=34)	Gratitude (GQ-6); depression (HADS); satisfaction (SWLS)	8 weeks
Lai et al.[42]	Participants (n=108)	Isolated	Gratitude diary (n=51)	Control (n=57)	Gratitude (GQ-6, GAC); affect (PANAS)	3 weeks
Mao et al.[43]	Students (n=205)	Isolated	High-gratitude priming (n=110)	Control (n=95)	Gratitude (GQ-6)	No follow-up
Martínez-Martí et al.[44]	Students (n=105)	Isolated	Gratitude intervention (n=41)	Hassle report (n=30) and no intervention (n=34)	Gratitude, affect, well-being, physical symptoms, pain, sleep, relationship quality, perception of the needs of others	2 weeks
Matvienko-Sikar et al.[45]	Pregnant women in prenatal care (n=46)	Combined	Gratitude diary and reflection audio (n=32)	Control (n=14)	Stress (PDQ and EPDS); gratitude (GDP); reflection (MAAS)	4.5 weeks
Ng et al.[46]	Adults with an average age of 20 years (n=212)	Isolated	Gratitude stimulation (n=107)	Control (n=105)	Gratitude and humor	No follow-up
	Adults with an average age of 31 years (n=331)	Isolated	Gratitude stimulation (n=111)	Stimulus of joy (n=110); without stimulation (n=110)	Gratitude, affect, and conformity	
O'Connell et al.[47]	College students (n=91)	Isolated	Gratitude diary (N=29) Interpersonal gratitude diary (n=30)	Control diary (n=32)	Gratitude (GQ-6); satisfaction (SWLS)	1 month
O'Connell et al.[48]	Adults with an average age of 22 years (n=192)	Isolated	Gratitude writing (n=47); writing and expressing gratitude (n=40)	Control (n=61)	Life satisfaction (SWLS); affect (SPANE); depression (CES-D 10); Gratitude (GQ-6); satisfaction in the relationship and expression of gratitude in relationships	1 or 3 months
Oishi et al.[49]	Students with an average age of 22 years (n=336)	Isolated	Gratitude writing (n=168)	No gratitude writing (n=168)	Happiness; satisfaction; pleasure; gratitude; appreciation; sadness; anger; indebtedness; shame and guilt	No follow-up
	Students with an average age of 20 years (n=218)	Isolated	Gratitude writing (n=101)	No gratitude writing (n=117)		
	Students with an average age of 18 years (n=181)	Isolated	Gratitude writing (n=85)	No writing (n=96)		
O'Leary et al.[50]	Women (n=61)	Isolated	Gratitude intervention (n=29)	Reflection intervention (n=22); waiting list (n=10)	Stress (PSS); depression (EDS); happiness (SHS)	5 weeks
Osborn et al.[51]	Students (mean age: 15 years) (n=103)	Combined	Shamiri-Digital (n=50)	Study abilities (n=53)	Depression (PHQ-8); anxiety (GAD-7); well-being (SWEM-WBS); happiness and optimism (EPOCH); viability and acceptance	2 weeks
Otsuka et al.[52]	Workers (n=76)	Isolated	Gratitude intervention (n=38)	Control (n=38)	Gratitude; affect (PANAS); life satisfaction (SWLS); happiness (SHS) and well-being	4 weeks
Otto et al.[53]	Patients with treated breast cancer (n=67)	Isolated	Gratitude stimulation (n=34)	Control (n=33)	Gratitude, positive affect, goal seeking, fear of recurrence and death	1 and 3 months
Ouweneel et al.[54]	Students with an average age of 21 years (n=50)	Isolated	Gratitude intervention (n=25)	Daily experience (n=25)	Emotions (JAWS); commitment (UWES–S)	5 weeks

continue...

...Continuation

Appendix B. Study characteristics

Author	Population	Type of intervention	Intervention	Comparison	Outcomes	Follow-up
Owens et al.[55]	Children aged 5 to 11 years (n=62)	Isolated	Gratitude intervention (n=22)	Best condition (n=23); Daily (n=17)	Affect (PANAS-C); satisfaction (BMSLSS); self-esteem (PCS-C)	1 weeks
Proyer et al.[56]	Women (n=163)	Isolated	Gratitude visits (n=30)	3 good things (n=44); 3 funny things (n=20); strengths (n=35); control (n=34)	Happiness (AHI); depression (CES-D)	6 meses
Ramírez et al.[57]	Elderly (n=56)	Combined	Program based on forgiveness, gratitude, and life review (n=28)	Control (n=28)	Anxiety (STAI); depression (Beck Depression Inventory, BDI); memory (AMT); cognition (MEC); satisfaction (LSS); happiness (SHS)	4 months
Redwine et al.[58]	Heart failure patients (n=70)	Isolated	Gratitude diary (n=34)	Usual care (n=36)	Gratitude (CQ-6); exercise activities (LTEQ)	8 weeks
Renshaw et al.[59]	College students (n=97)	Isolated	Gratitude thoughts (n=54)	Control (n=43)	Gratitude (GQ-6); satisfaction (SWLS); happiness (SHS); affect (PANAS)	2 weeks
Riskin et al.[60]	43 teams of neonatal ICU workers (n=163)	Isolated	Expression of gratitude by physicians and mothers (n=40), only mothers (n=42), only physicians (n=43)	Neutral expression (n=38)	Team performance (diagnosis, treatment, sharing)	No follow-up
Roth et al.[61]	Children (n=42)	Combined	Positive psychology intervention (n=21)	Control (n=21)	Satisfaction (BMSLSS); affect (PANAS)	10 weeks + 2 months
Schache et al.[62]	Teenagers with *diabetes mellitus* type 1 (n=80)	Isolated	Daily gratitude record (n=40)	Usual care (n=40)	Stress (DSQY); depression (CESDSC); quality of life (DQLYS); self-care (SCIRV); gratitude (GQ-6)	12 weeks
Shin et al.[63]	Participants (n=630)	Isolated	Letters of gratitude to parents (n=305)	Control (n=325)	Affect (PANAS); Gratitude (GAC)	2 weeks
Tan et al.[64]	Adults with stage III or IV cancer (n=92)	Isolated	Gratitude diary (n=49)	Control diary (n=43)	Gratitude (GQ-6)	1 week
Toepfer et al.[65]	Participants (n=219)	Isolated	Gratitude intervention (n=141)	Control (n=78)	Gratitude (GQ-6); satisfaction (SWLS); happiness (SHS); depression (CES-10)	4 weeks
Tofangchi et al.[66]	Participants (n=32)	Isolated	Gratitude intervention (n=16)	Control (n=16)	Happiness (OHQ)	40 days
Vayness et al.[67]	People with an average age of 19 years (n=96)	Isolated	Induction of gratitude emotion (n=32)	Induction of happiness (n=32) and neutral (n=32)	Happiness and gratitude	No follow-up
	Workers with an average age of 35 years (n=143)	Isolated	Induction of gratitude emotion (n=64)	Induction of happiness (n=42) and neutral (n=37)	Happiness and gratitude	No follow-up
Vogelsang et al.[68]	Participants (n=169)	Isolated	Gratitude intervention (n=85)	Control (n=84)	Gratitude (numeric scale)	6 weeks
Völler et al.[69]	Participants (n=118)	Isolated	Gratitude intervention (n=51)	Control (n=67)	Stress (PSS); well-being (MHC-SF)	12 weeks
Williams et al.[70]	Students (n=70)	Isolated	Gratitude expression (n=30)	Control (n=40)	Appreciation; interpersonal warmth; capacity of expression; affiliation intention	No follow-up
Wong et al.[71]	Psychotherapy patients (n=293)	Isolated	3 sessions of gratitude writing (n=127)	Expression writing (n=91); psychotherapy only (n=75)	Well-being, psychological symptoms, life function (BHM-20 and GMH); negative and positive emotions (LIWC)	12 weeks
Yang et al.[72]	Chinese prisoners (n=144)	Isolated	Gratitude intervention (n=48)	Kindness intervention (n=48); no intervention (n=48)	Affect (ABS); satisfaction (SWLS); well-being (IWB); subjective vitality (RFS)	6 weeks

Appendix C. Risk of bias summary for the included studies

Author	Randomization	Allocation Concealment	Double blinding	Evaluators blinding	Losses (<20%)	Prognostic characteristics	Appropriate outcomes	ITT	Sample calculation	Early interruption
Emmons et al.[1]	?	?	-	?	?	?	+	?	?	?
Antoine et al.[10]	?	?	-	?	+	+	+	+	-	+
Baker et al.[11]	?	?	-	?	?	?	+	?	-	?
Bartlett et al.[12]	-	-	+	?	?	?	+	?	?	+
Bohlmeijer et al.[13]	+	+	-	?	-	+	+	-	+	+
Cheng et al.[14]	+	+	-	?	+	+	+	+	+	+
Cunha et al.[15]	+	+	-	?	-	?	+	-	+	+
Datu et al.[16]	-	-	-	?	+	-	+	-	-	+
Dennis et al.[17]	+	+	-	?	+	+	+	+	+	+
DeSteno et al.[18]	?	?	-	?	?	?	+	?	?	?
Ducasse et al.[19]	+	+	-	+	+	+	+	-		
Froh et al.[20]	?	?	-	?	?	+	+	?	-	?
Gavian et al.[21]	+	+	-	?	+	+	+	-	+	?
Geraghty et al.[22]	+	+	-	?	-	?	+	-	-	?
Grant et al.[23]	?	?	-	?	?	?	+	?	?	?
Gulliford et al.[24]	?	?	-	?	-	-	+	-	-	+
Heckendorf et al.[25]	+	+	-	?	-	+	+	+	+	+
Heekerens et al.[26]	+	+	-	?	+	-	+	+	-	+
Heintzelman et al.[27]	?	?	-	?	-	?	+	+	+	+
Hirshberg et al.[28]	+	+	-	?	+	+	+	-	+	+
Ho et al.[29]	+	+	-	?	?	+	+	?	-	+
Hülsing et al.[30]	?	?	-	?	-	+	+	-	-	+
Hussong et al.[31]	?	?	-	?		?	+	?	?	+
Jackowska et al.[32]	+	+	-	?	?	+	+	?	-	+
Kanagawa et al.[33]	?	?	-	?	+	+	+	+	+	+
Karns et al.[34]	?	?	-	?	+	+	+	?	-	+
Ki et al.[35]	?	?	-	?	?	?	+	?	-	?
Kini et al.[36]	?	?	-	?	?	+	+	?	-	+
Kloppenborg et al.[37]	+	+	-	?	+	+	+	+	+	+
Koay et al.[38]	?	?	-	?	+	-	+	+	-	+
Kobayashi et al.[39]	?	?	-	?	+	-	+	+	-	+
Krentzman et al.[40]	?	?	-	?	?	?	+	?	?	?
Kwok et al.[41]	?	?	-	?	+	+	+	+	-	+
Lai et al.[42]	?	?	-	?	+	+	+	+	-	+
Mao et al.[43]	?	?	-	?	+	-	+	+	+	+
Martínez-Martí et al.[44]	?	?	-	?	?	?	+	?	?	?
Matvienko-Sikar et al.[45]	+	+	-	?	-	+	+	-	+	+
Ng et al.[46]	?	?	-	?	?	?	+	?	+	+
O'Connell et al.[47]	+	+	-	?	-	+	+	+	+	+
O'Connell et al.[48]	+	+	+	?	+	+	+	-	-	+
Oishi et al.[49]	?	?	-	?	?	?	+	?	+	+
O'Leary et al.[50]	?	?	-	?	?	?	+	?	?	?
Osborn et al.[51]	+	+	-	?	+	+	+	+	-	+
Otsuka et al.[52]	?	?	-	?	?	?	+	?	-	
Otto et al.[53]	?	?	-	?	?	?	+	?	-	+
Ouweneel et al.[54]	?	?	-	?	?	+	+	?	?	?
Owens et al.[55]	?	?	-	?	?	?	+	?	?	?
Proyer et al.[56]	?	?	-	?	?	+	+	?	+	?

continue...

...Continuation

Appendix C. Risk of bias summary for the included studies

Author	Randomization	Allocation Concealment	Double blinding	Evaluators blinding	Losses (<20%)	Prognostic characteristics	Appropriate outcomes	ITT	Sample calculation	Early interruption
Ramírez et al.[57]	?	?	-	?	?	?	+	?	?	?
Redwine et al.[58]	+	+	-	?	-	+	+	-	+	+
Renshaw et al.[59]	+	+	-	?	?	+	+	?	?	+
Riskin et al.[60]	+	?	-	?	?	+	-	?	+	+
Roth et al.[61]	+	+	-	?	+	+	+	+	?	+
Schache et al.[62]	+	+	-	?	-	+	+	+	+	+
Shin et al.[63]	?	?	-	?	+	+	+	-	?	+
Tan et al.[64]	+	+	-	?	+	+	+	+	+	+
Toepfer et al.[65]	?	?	-	?	?	?	+	?	-	?
Tofangchi et al.[66]	?	?	-	?	?	?	-	?	-	?
Vayness et al.[67]	?	?	-	?	-	?	+	-	+	+
Vogelsang et al.[68]	+	+	-	?	-	+	+	-	?	+
Völler et al.[69]	?	?	-	?	+	+	+	+	?	+
Williams et al.[70]	?	?	-	?	?	?	+	?	?	?
Wong et al.[71]	?	?	-	?	-	?	+	+	-	+
Yang et al.[72]	-	-	-	?	?	+	+	?	-	+

Note. +: High risk; –: Low risk; ?: Unclear risk; ITT: intention-to-treat analysis.
